# Effect of Leaf Water Potential on Internal Humidity and CO_2_ Dissolution: Reverse Transpiration and Improved Water Use Efficiency under Negative Pressure

**DOI:** 10.3389/fpls.2017.00054

**Published:** 2017-02-06

**Authors:** Timo Vesala, Sanna Sevanto, Tiia Grönholm, Yann Salmon, Eero Nikinmaa, Pertti Hari, Teemu Hölttä

**Affiliations:** ^1^Department of Physics, University of HelsinkiHelsinki, Finland; ^2^Department of Forest Sciences, University of HelsinkiHelsinki, Finland; ^3^Viikki Plant Science Centre, University of HelsinkiHelsinki, Finland; ^4^Earth and Environmental Sciences Division, Los Alamos National LaboratoryLos Alamos, NM, USA

**Keywords:** water potential, CO_2_ assimilation, carbon uptake, water uptake, Kelvin effect, water use efficiency, redwood

## Abstract

The pull of water from the soil to the leaves causes water in the transpiration stream to be under negative pressure decreasing the water potential below zero. The osmotic concentration also contributes to the decrease in leaf water potential but with much lesser extent. Thus, the surface tension force is approximately balanced by a force induced by negative water potential resulting in concavely curved water-air interfaces in leaves. The lowered water potential causes a reduction in the equilibrium water vapor pressure in internal (sub-stomatal/intercellular) cavities in relation to that over water with the potential of zero, i.e., over the flat surface. The curved surface causes a reduction also in the equilibrium vapor pressure of dissolved CO_2_, thus enhancing its physical solubility to water. Although the water vapor reduction is acknowledged by plant physiologists its consequences for water vapor exchange at low water potential values have received very little attention. Consequences of the enhanced CO_2_ solubility to a leaf water-carbon budget have not been considered at all before this study. We use theoretical calculations and modeling to show how the reduction in the vapor pressures affects transpiration and carbon assimilation rates. Our results indicate that the reduction in vapor pressures of water and CO_2_ could enhance plant water use efficiency up to about 10% at a leaf water potential of −2 MPa, and much more when water potential decreases further. The low water potential allows for a direct stomatal water vapor uptake from the ambient air even at sub-100% relative humidity values. This alone could explain the observed rates of foliar water uptake by e.g., the coastal redwood in the fog belt region of coastal California provided the stomata are sufficiently open. The omission of the reduction in the water vapor pressure causes a bias in the estimates of the stomatal conductance and leaf internal CO_2_ concentration based on leaf gas exchange measurements. Manufactures of leaf gas exchange measurement systems should incorporate leaf water potentials in measurement set-ups.

## Introduction

Water potential is negative in the xylem of virtually all terrestrial plants (Pockman et al., 1995). Water potential is lowered by transpiration from the leaves assisted by the cohesive forces between water molecules causing water to be under tension, i.e., under negative pressure. According to Young-Laplace's formula (e.g., Nobel, 2005), the cohesive forces are balanced by the surface tension and the balance is manifested as curved, concave air-water surfaces in leaves. The higher the tension is, i.e., the lower the water potential, the stronger the concavity is. In addition to transpiration, solutes dissolved in the xylem sap may contribute to the decrease in xylem water potential. However, the osmotic component of water potential in the apoplastic water is marginal since the amount of dissolved solutes in the apoplast is typically very small (Nobel, 2005) and we ignore the osmotic effect here.

The negative water potential causes the vapor pressure of water in the sub-stomatal cavities to be lowered in relation to the vapor pressure of water at a water potential of zero, i.e., pure water under atmospheric pressure (Pickard, 1981; Nobel, 2005). The reduction in the vapor pressure can be also derived from the effect the concavity of water surface makes. Over the concave surface the equilibrium vapor pressure of water is lowered from that over a flat surface. This is called the Kelvin effect (see Appendix A). The former approach, based on the concept of the potential, is used by plant scientists, whereas the latter one, based on the curvature effect, is known by physicists. These two approaches seem to be different, but as the water potential is coupled with the surface curvature via Young-Laplace's equation, they are actually one and the same leading to the following phenomenon: The water vapor concentration in the internal (sub-stomatal/intercellular) cavity is lower than that corresponding to the relative humidity (RH) of 100% of the ambient air. The phenomenon is applied for example in the measurement of xylem water potential by the psychrometric technique (e.g., Boyer, 1968).

The significance of the vapor pressure decrease to leaf gas exchange and its implications for possible foliar water uptake by “reverse transpiration”, i.e., direct stomatal water vapor intake, have remained unexplored in plant physiology. The reduction of water vapor pressure at the site of evaporation in the leaves should have significant consequences for plants, but only at low leaf water potentials and at high RH. It is well-recognized that foliar water uptake occurs, and its importance is especially pronounced during water stress, in the air of high humidity and in trees living in fogbelt regions (Breazeale et al., 1950; Haines, 1952; Burgess and Dawson, 2004; Breshears et al., 2008; Limm et al., 2009). An example of such is the coastal redwood [*Sequoia sempervirens* (D. Don) Endl.], living in the coastal Californian forests, which relies heavily on the regular fog deposition for up to 30% of their yearly water uptake (Burgess and Dawson, 2004). Water acquisition from ambient air may also allow the coastal redwood to reach heights of over 100 m (Koch et al., 2004). However, the reverse transpiration does not rule out the fog-droplet-mediated water uptake. It seems that many of the plant species demonstrating foliar water uptake are not from frequently foggy environments and simply having periods of leaf wetness may be a primary prerequisite for the uptake (Berry et al., 2014). Since the vapor pressure decrease modifies the driving force (vapor pressure difference) of the transpiration, it has also implications for the estimation of stomatal conductance from leaf gas exchange measurements.

The curvature does not modify only the equilibrium vapor pressure of water but the pressures of all dissolved gases, like CO_2_. This has remained almost unrecognized by the plant science community although it is well-known in e.g., atmospheric physics [however see Schenk et al. (2016) for the role of the same effect for the dissolution of gases in xylem sap]. Namely, if the air-water surfaces are concave, the equilibrium vapor pressure of CO_2_ is reduced, i.e., its effective solubility to the water phase increases (Vehkamäki, 2006; see also Lewis and Randall, 1961; Vesala et al., 1997; Rodriguez-Navarro et al., 2002; Mercury et al., 2003; Pera-Titus et al., 2009). The decrease in the saturation vapor concentration of CO_2_ above the water-air menisci inside the leaf increases the partitioning of CO_2_ from the air phase to the aqueous phase at the air/water interface. This further decreases leaf internal CO_2_ concentration and enhances CO_2_ transport and assimilation rates. These together with the reduction in transpiration improve the instantaneous water use efficiency. The increased partitioning to the aqueous phase should be important especially under high light, when the photosynthetic production of plants is most limited by the diffusion rate of CO_2_ from the ambient atmosphere to the chloroplasts of the mesophyll cells (Aalto and Juurola, 2002).

Even if plants would not be water-limited, the enhanced CO_2_ solubility benefits them as the enhanced carbon uptake increases water use efficiency. At arid environments, where low soil and leaf water potentials are common the water use efficiency is enhanced both by reduced water and CO_2_ equilibrium vapor pressures. If these conditions are combined with e.g., high nocturnal RH levels, plant water status can be further improved by water vapor uptake from the air (reversed transpiration), as a result of the Kelvin effect. However, when leaves are wet the stomata, even if open, may be blocked with a film of water on the cuticle. This would block the CO_2_ uptake but not the reversed transpiration if the surface tension prevents the cuticular film on penetrating into a sub-stomatal cavity. Furthermore, the low water potential would hinder the formation of the sub-stomatal water film by drawing the water into cell walls or xylem. Then the reverse transpiration would still occur by means of evaporation from the cuticular water film crossing a stomatal pore and subsequent condensation on the mesophyll surface.

In this study we use theoretical considerations and model calculations to evaluate the significance of the reduced vapor pressure of water and CO_2_ for plant leaf gas exchange. We also quantify its contribution to plant water use efficiency. We demonstrate that water uptake in vapor form by reverse transpiration would be theoretically sufficient to explain the observed rates of foliar water uptake in coastal redwood trees.

## Materials and methods

### Reduced water vapor pressure and transpiration

The ratio of the saturation water vapor concentration (*w*_*i*_) (or saturation water vapor pressure; for an ideal gas these are interchangeable) over a liquid-gas interface to that over an interface with water potential of zero (*w*_*i*,0_) is (Nobel, 2005)

(1) $$\frac{w_{i}}{w_{i,0}}=\exp\left(\frac{\psi V_{H2O}}{RT}\right)$$

where ψ is the water potential, *V*_*H*20_ is the molar volume of water (18 × 10^−6^ m^3^ mol^−1^), *R* is the universal gas constant and *T* the interfacial temperature (for various symbols see Table 1). In case of water at water potential of zero, i.e., pure water at atmospheric pressure, the ratio is one and the saturation vapor concentration (*w*_*i*,0_) depends only on temperature (Nobel, 2005).

**Table 1:** **Symbols and physical constants used in the calculations**.

**Symbol**	**Meaning**	**Units/Value (if applicable)**
*A*	CO_2_ assimilation rate	mol m^−2^s^−1^^***^
*A_0_*	CO_2_ assimilation rate in case of water potential 0	mol m^−2^s^−1^^***^
*c*_*aq*_	Concentration of CO_2_ in aqueous phase	mol m^−3^
*c*_*aq*,0_	Concentration of CO_2_ in aqueous phase in case of water potential 0	mol m^−3^
*c*_*a*_	CO_2_ concentration in ambient air	mol m^−3^
*c*_*i*_	Internal (sub-stomatal/intercellular) CO_2_ concentration	mol m^−3^
*c*_*i*,*app*_	Apparent internal (sub-stomatal/intercellular) CO_2_ concentration	mol m^−3^^*****^
*c*_*c*_	CO_2_ concentration at the chloroplast	mol m^−3^
*D*	Diffusion coefficient of water vapor in air	2.4·10^−9^ m^2^ s^−1^^****^
*E*	Transpiration rate	mol m^−2^s^−1^^***^
*E_0_*	Transpiration rate in case of water potential 0	mol m^−2^s^−1^^***^
*f*	A constant of proportionality between chloroplast CO_2_ concentration and CO_2_ assimilation rate	m^3^ m^−2^s^−1^^***^
*g*	Stomatal conductance	ms^−1^^***^
*g_*app*_*	Apparent stomatal conductance	ms^−1^^*****^
$g_{co_{2}}^{air}$	Air phase diffusive conductance from ambient air to the sub-stomatal cavity	m s^−1^^***^
$g_{co_{2}}^{aq}$	Aqueous phase diffusive conductance from the sub-stomatal cavity to the chloroplast	m s^−1^^***^
*H_*CO*2_*	Henry's law coefficient for CO_2_	Unitless; the ratio of the gas phase CO_2_ concentration to that in the liquid phase at the equilibrium ^****^
$j_{co_{2}}^{air}$	Flux rate of CO_2_ between the ambient air and sub-stomatal cavity	mol m^−2^ s^−1^^***^
$j_{co_{2}}^{aq}$	Flux rate of CO_2_ between the sub-stomatal cavity and chloroplast	mol m^−2^ s^−1^^***^
*r*	Radius of curvature	m^*^
*R*	Universal gas constant	8.314 J K^−1^ mol^−1,^^****^
*S*	Saturation ratio (relative humidity / 100%)	–
*T*	Temperature	K
*T_*N*_*	Water tension	Pa^**^
*V*_*H*2*O*_	Molar volume of water	18·10^−6^ m^3^ mol^−1,^^****^
*V*_*CO*2_	Partial molar volume of CO_2_ in water	34·10^−6^ m^3^ mol^−1,^^****^
*w*_*i*_	Internal (sub-stomatal/intercellular) water vapor concentration	mol m^−3^
_*w*_*i*_,0_	Internal (sub-stomatal/intercellular) water vapor concentration in case of water potential 0	mol m^−3^
*w*_*a*_	Water vapor concentration in the ambient air	mol m^−3^
γ	Surface tension of water	0.073 N m^−1,^^****^
ψ	Water potential	Pa

* *Positive radius of curvature is concave, negative radius of curvature is convex*.

** Tension can be expressed in terms of water potential (ψ): For pure water T_N_ = − ψ

*** *Expressed here per leaf area. The aqueous phase diffusive conductance consists also of lipid components*.

**** Reference from CRC handbook of chemistry and physics. 2001. At a temperature of 18°C

***** *Apparent means that it is estimated from leaf gas exchange measurements without taking into account the effect the changes in water pressure due to lowered water potential*.

Transpiration rate (*E)*, is proportional to the difference between the water vapor concentration in the sub-stomatal cavity *w*_*i*_ and water vapor concentration in the ambient air *w*_*a*_

(2) $$\begin{array}{r} E=\left(w_{i}-w_{a}\right)g \end{array}$$

where *g* is stomatal conductance (Nobel, 2005). Inserting Equation (1) into Equation (2) and introducing the saturation ratio of the ambient air ($S\equiv \frac{w_{a}}{w_{i,0}}$; RH being 100% × *S*) the transpiration rate is expressed as

(3) $$\begin{array}{l} E=\left(w_{i,0}\exp\left(\frac{\psi V_{H2O}}{RT}\right)-w_{a}\right)g\\ \;=w_{i,0}\left(\exp\left(\frac{\psi V_{H2O}}{RT}\right)-S\right)g \end{array}$$

For the flat surface exp*(*ψ*V*_*H*2*O*_*/RT)* = *1* and for negative water potentials the term is less than one (see **Figure 2**) and that facilitates the situation that the transpiration (*E*) turns to negative when *S* > exp*(*ψ*V*_*H*2*O*_*/RT)*. The actual transpiration rate in relation to the transpiration rate at a leaf water potential of zero (*E*_0_) is then

(4) $$\begin{array}{l} \frac{E}{E_{0}}=\frac{w_{i,0}\left(\exp\left(\frac{\psi V_{H2O}}{RT}\right)-S\right)g\;}{(w_{i,0}-w_{a})g}=\frac{\left(\exp\left(\frac{\psi V_{H2O}}{RT}\right)-S\right)g\;}{(\frac{w_{i,0}}{w_{i,0}}-\frac{w_{a}}{w_{i,0}})g}\\ \;=\frac{\exp\left(\frac{\psi V_{H2O}}{RT}\right)-S}{1-S} \end{array}$$

Also boundary layer conductance contributes to leaf water exchange, but here it is included in the stomatal conductance term g.

### Reduced vapor pressure of CO_2_ and CO_2_ assimilation rate and the water-use efficiency

Saturation vapor concentration of CO_2_ over a surface of water at water potential of zero is commonly expressed using Henry's law

(5) $$\begin{array}{r} c_{aq,0}=c_{i}H_{CO2} \end{array}$$

where *c*_*i*_ and *c*_*aq*,0_ are the leaf CO_2_ concentrations in the air and aqueous phases, and *H*_*CO*2_ is the Henry's law coefficient for CO_2_ in water, which is dependent on temperature and pH (Nobel, 2005). If the decrease in water potential at the site of evaporation is caused solely by loss of water (and not osmotic concentration) then the Henry's law's relation in Equation (5) is modified so that (Vehkamäki, 2006)

(6a) $$c_{aq\;=\;c_{i}}H_{CO2}\exp\left(-\frac{2\gamma V_{CO2}}{rRT}\right)\;=\;c_{i}H_{CO2}\;\exp\left(-\frac{T_{N}V_{CO2}}{RT}\right)$$

where γ is surface tension of water, *r* is the radius of curvature (defined positive for a concave surface), *V*_*CO*2_ is the partial molar volume of CO_2_ in water (34 × 10^−6^ m^3^ mol^−1^), *T*_*N*_ is the water pressure difference over the air-water interface (T_N_ = P_air_ − P_liquid_, where P_air_ is air pressure and P_liquid_ is liquid pressure), and where the Young-Laplace equation (T_N_ = 2γ*/r*) is used for the relation between interface curvature and surface tension. Note that in physics *r* is typically defined negative for a concave surface but we follow here the convention often used in eco-physiology of plants. Equation (6a) means that the higher the T_N_, the lower the saturation vapor pressure, and hence the larger the partitioning to the aqueous phase (the larger “effective Henry's law constant,” *H*_*CO*2_ times the exponential term) of CO_2_ in water. Since the osmotic concentration of the apoplastic water is generally assumed to be small (Nobel, 2005), we assume in the following calculations that the water potential at air-water interface is lowered only by T_N_ and the osmotic component of water potential is negligible, i.e., −ψ = 2γ*/r*, leading to:

(6b) $$\begin{array}{r} c_{aq}=c_{i}H_{CO2}\;\exp\left(\frac{\psi V_{CO2}}{RT}\right) \end{array}$$

The driving force for stomatal gas exchange of CO_2_ is the difference between the CO_2_ concentration in ambient air and the CO_2_ concentration at the site of photosynthesis in the chloroplasts *c*_*c*_. The pathway of CO_2_ movement is divided into air and aqueous phases. In steady state, CO_2_ flux in the air phase $\;\left(J_{CO2}^{air}\right)$

(7) $$\begin{array}{r} J_{CO2}^{air}=\left(c_{a}-c_{i}\right)g_{CO2}^{air} \end{array}$$

must equal the aqueous phase flux $\left(J_{CO2}^{aq}\right)$

(8) $$\begin{array}{r} J_{CO2}^{aq}=\left(c_{aq}-c_{c}\right)g_{CO2}^{aq} \end{array}$$

where $g_{CO2}^{air}$ and $g_{CO2}^{aq}$ are the air and aqueous phase diffusive conductances, respectively. Both fluxes must also equal the net assimilation rate (*A*) in steady state, which we approximate to be linearly proportional to CO_2_ concentration in the chloroplasts (e.g., Mäkelä et al., 1996)

(9) $$\begin{array}{r} A=fc_{c} \end{array}$$

where *f* is a constant of proportionality depending on light availability, temperature, and biochemical properties of the photosynthetic machinery. The relation between CO_2_ assimilation rate and chloroplasts is assumed linear in order that an analytical solution can be obtained for assimilation rate as a function of leaf water potential. Note that in reality, *A* saturates with increasing *c*_*c*_ (this becomes more evident at higher CO_2_ concentrations), and also the compensation point of CO_2_ is involved in the classical Farquhar formulation of the photosynthesis rate (Von Caemmerer and Farquhar, 1981). However, Equation (9) is a reasonable assumption over any small range of *c*_*c*_ and over the CO_2_ limited region of the A–c_c_ curve (Lambers et al., 2008).

Combining equations (6b) to (9) reveals that the relative increase in CO_2_ assimilation rate resulting from the reduced vapor pressure of CO_2_ can be expressed simply in terms of the ratio between internal (air phase) and ambient CO_2_ concentration (see Supplementary Material for the algebraic derivation)

(10) $$\frac{A}{A_{0}}={\left[\frac{c_{i}}{c_{a}}\left(\frac{1}{\exp\left(\frac{\psi V_{CO2}}{RT}\right)}-1\right)+1\right]}^{-1}$$

This formulation shows that the values for the resistances to CO_2_ diffusion, light level and the absolute value of the Henry's law coefficient need not to be considered to evaluate the relative effect of water potential on the CO_2_ assimilation rate. Further calculations (see Supplementary Material) reveal that the time scale required for the steady state CO_2_ concentration to be reached in the leaves after changes in water potential is less than 1 s so the steady state approach used here is well-justified.

Note that Equation (10) does not contain the effective Henry's law coefficient *H*_*CO*2_ including the dissociation of CO_2_ into ${\text{HCO}}_{3}^{-}$ at higher pH values (Taiz and Zeiger, 2002), and the aqueous phase diffusive conductance $g_{CO2}^{aq}$. They are quantities of which estimation requires information beyond the modeling framework here. The aqueous pathway of CO_2_ from the mesophyll surface into the chloroplast stroma is more complex than portrayed here, affected by e.g., lipid phase diffusive steps or different macromolecules obstructing diffusion (Taiz and Zeiger, 2002; Nobel, 2005).

The final note is related to assuming osmotic effects negligible. For dilute solutions the equilibrium water vapor pressure is lowered according to the Raoult's law, i.e., the equilibrium pressure of the pure water must be multiplied by the mole fraction of water in the solution. This creates negative water potential facilitating the negative transpiration similarly to a non-flat surface. Then $w_{i}=w_{i,0}X_{H2O}\exp\left(-\frac{2\gamma V_{H2O}}{rRT}\right)$ (compare with Equation 1) where *X*_*H*2*O*_ is the mole fraction of water in the solution and is less than one. For CO_2_ the situation is more complicated and one cannot say generally whether the osmotic effect lowers or increases the equilibrium vapor pressure. This depends on the type of solutes and their interactions with the dissolved CO_2_ and water molecules.

**Figure 1:**
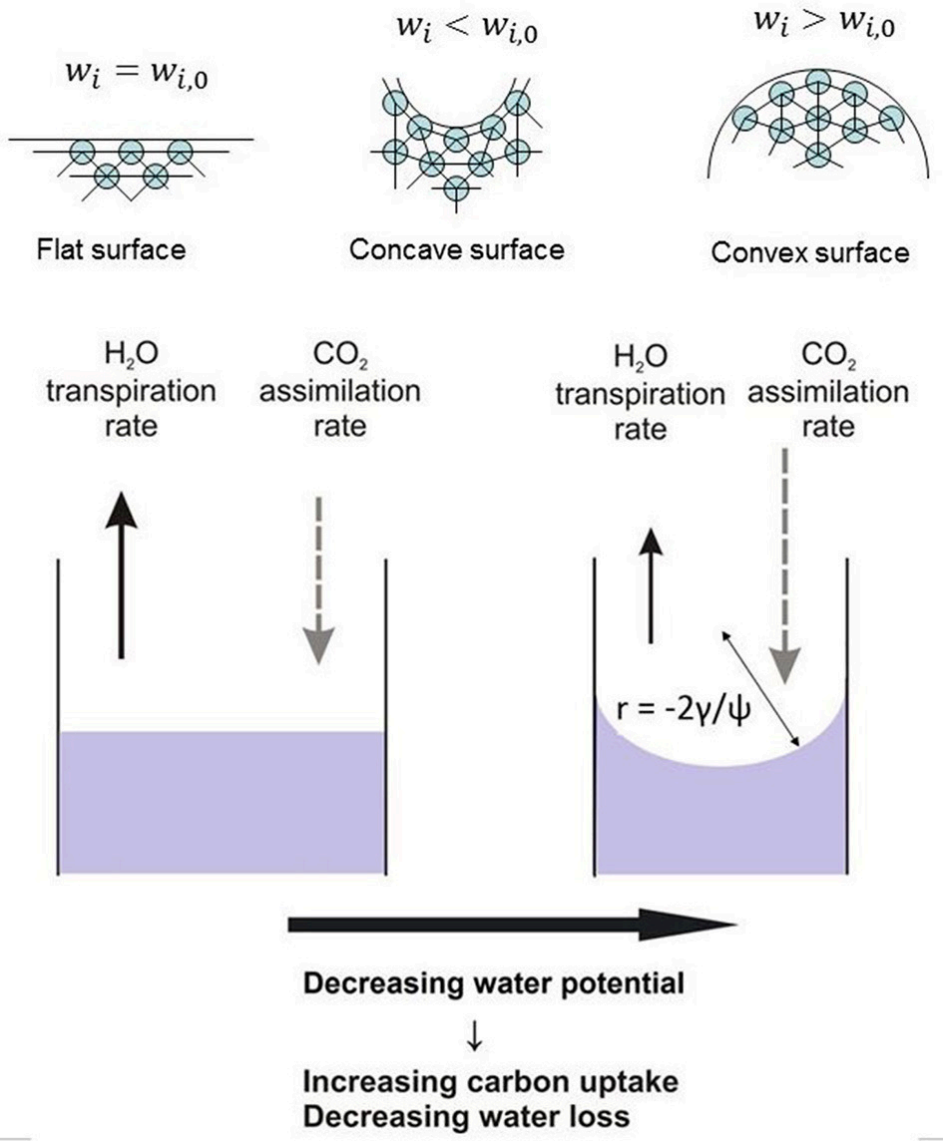
**Over a curved water/air meniscus at the air-mesophyll interface the equilibrium vapor concentrations for both water vapor and CO_**2**_ are lowered**. Consequently, the exchange rates of water vapor and CO_2_ are affected. The radius of curvature *r* of the meniscus is given by the Young-Laplace equation linking the surface tension γ and water potential ψ.

Figure 1 concludes schematically the consequences of the existence of the curved interface for transpiration and carbon assimilation.

Finally, we define the water-use efficiency (*WUE*) as the ratio of the net assimilation and the transpiration rate as

(12) $$\begin{array}{r} WUE=\frac{A}{E} \end{array}$$

### Effects of the reduced vapor pressures on the stomatal conductance and the internal CO_2_ concentration

Ignoring the reduction of vapor pressure with decreasing water potential will also cause an error in the estimation of the value of stomatal conductance and leaf internal CO_2_ concentrations from measurements of leaf gas exchange and vapor pressure deficit (VPD), especially when the measurements are conducted at high RH and low xylem water potential. Using Equation 3 for the actual stomatal conductance (*g*) and introducing the apparent stomatal conductance (*g*_*app*_), i.e., the stomatal conductance if the reduction in water vapor pressure is ignored

(12) $$g=\frac{E}{w_{i,0\;}\exp\left(\frac{\psi V_{H2O}}{RT}\right)-w_{a}}$$

(13) $$g_{app}=\frac{E}{w_{i,0}-w_{a}}$$

the relationship between two conductances can be written as

(14) $$\begin{array}{l} g=\frac{\frac{E}{w_{i,0\;}\exp\left(\frac{\psi V_{H2O}}{RT}\right)-w_{a}}}{\frac{E}{w_{i,0}-w_{a}}}g_{app}=\frac{w_{i,0}-w_{a}}{w_{i,0\;}\exp\left(\frac{\psi V_{H2O}}{RT}\right)-w_{a}}g_{app}\\ \;=\frac{w_{i,0}(1-S)}{w_{i,0}\left(\exp\left(\frac{\psi V_{H2O}}{RT}\right)-S\right)}g_{app}=\frac{1-S}{\exp\left(\frac{\psi V_{H2O}}{RT}\right)-S}g_{app} \end{array}$$

The error made in the estimation of stomatal conductance will also propagate into the calculation of the internal CO_2_ concentration (*c*_*i*_). Formulating the CO_2_ exchange by means of the two conductances as

(15a) $$c_{a}-c_{i}=\frac{A}{g}$$

(15b) $$c_{a}-c_{i,app}=\frac{A}{g_{app}}$$

where c_i,app_ is the internal CO_2_ concentration, the ratio of the difference of the ambient CO_2_ concentration and the internal CO_2_ concentration to the difference against the apparent internal CO_2_ concentration can be written as

(16) $$\frac{c_{a}-c_{i}}{c_{a}-c_{i,app}}=\frac{\frac{A}{g}}{\frac{A}{g_{app}}}=\frac{g_{app}}{g}=\frac{\exp\left(\frac{\psi V_{H2O}}{RT}\right)-S}{1-S}\;$$

## Results

### Effects of reduced vapour pressure on transpiration, assimilation, and water-use efficiency

**Figure 2:**
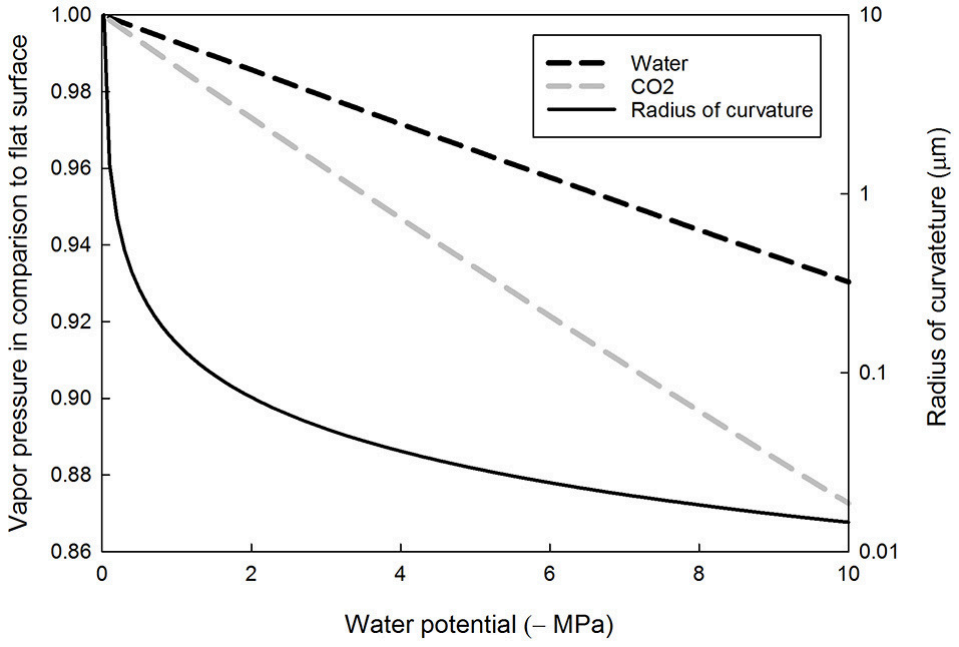
**Equilibrium vapor pressure of liquid water (black dashed line) and dissolved carbon dioxide (gray dashed line), normalized to the flat surface value, and the radius of curvature of the water meniscus as a function of the absolute value of the water potential**. The radius of concave surface is positive.

Decreasing leaf water potential decreases the saturation vapor concentration of both water and CO_2_, albeit more so in the latter than the former (Figure 2). This is because the partial molar volume of CO_2_ in the water solution is larger than that of the molar volume of water (see Table 1). Decreasing water potential lowers the CO_2_ saturation vapor pressure resulting in enhanced partitioning of CO_2_ to water. For example, at water potential of −0.5, −2.0, and −10.0 MPa, the partitioning of CO_2_ to water is 100.6, 102.8, and 114.6%, respectively, compared to that at water potential of zero, based on Equation (6b). Typical values of leaf water potential for C_3_ plants are between −1 and −2 MPa (Mencuccini, 2003), down to −4 MPa in species in arid zones, and as low as −10 MPa in the most extreme cases (Tyree, 1997).”

**Figure 3:**
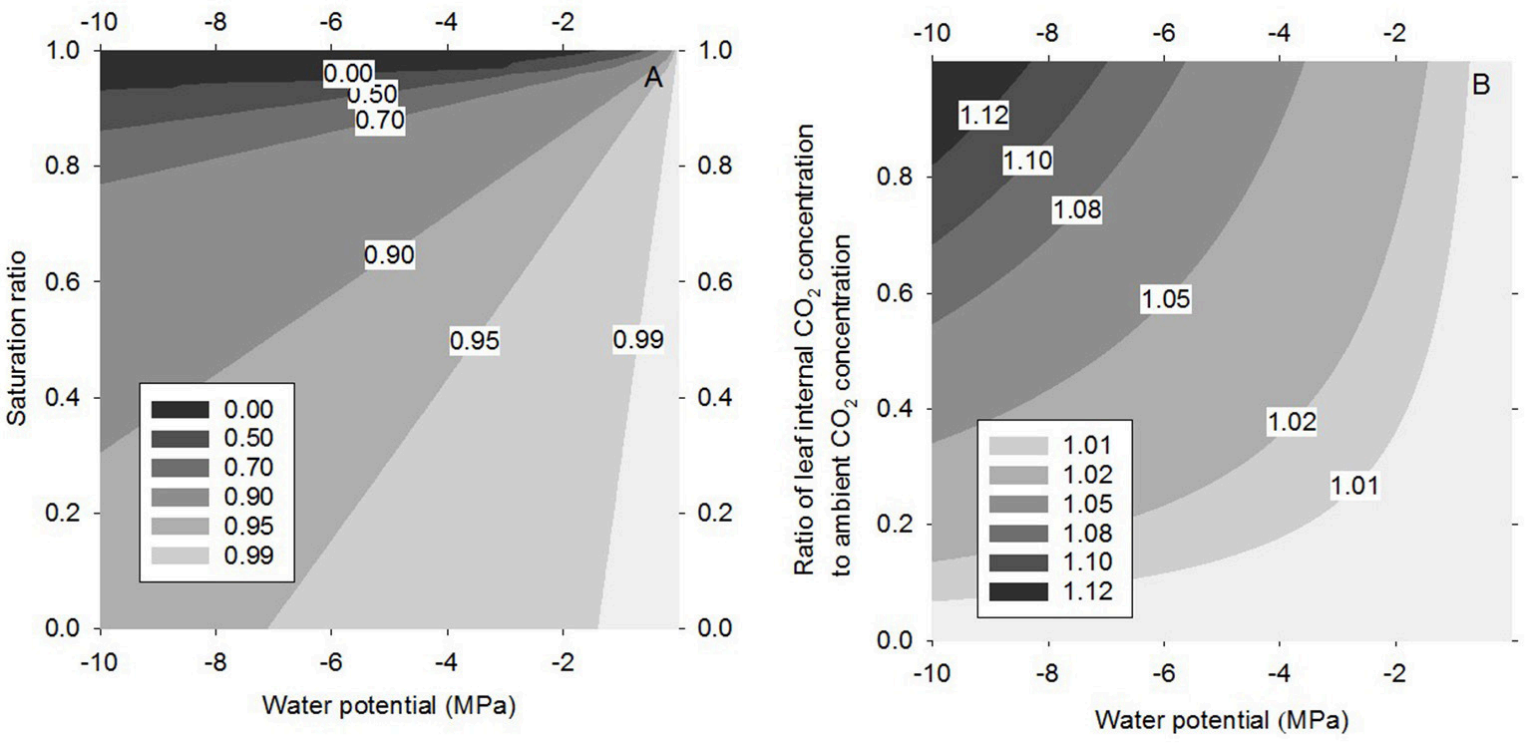
**Transpiration rate as a function of the saturation ratio ***S*** (***S*** = RH/100%) and the water potential (A)** and the CO_2_ assimilation rate as a function of the CO_2_ concentration in the internal (sub-stomatal/intercellular) cavity and the water potential **(B)**, both gas exchange rates normalized to the flat surface value.

The transpiration rate decreases as the leaf water potential decreases and the ambient RH increases. In the case that water potential is very low and RH is very high transpiration is predicted to turn into water uptake (see Equation 4). The “reverse transpiration” occurs along the zero contour in Figure 3A, being approximately at 94% and −10 MPa, and 98.9% at −2 MPa.

The increase in CO_2_ assimilation rate with decreasing water potential becomes more pronounced with increasing internal CO_2_ concentration (Figure 3B), i.e., under conditions of high stomatal conductance and/or low light. The daytime ratio of internal to ambient CO_2_ concentration varies typically from 0.5 to 1 (e.g., Steudle, 2001), and even at low internal concentrations CO_2_ (half of the ambient one), CO_2_ assimilation rate increases by 1.7 and 8.7% for water potentials of −2.0 and −10 MPa, respectively. At water potentials of −10 MPa many plants will close their stomata (e.g., Pockman and Sperry, 2000). Naumburg et al. (2004) have reported non-zero stomatal conductances down to −7 MPa and Garcia-Forner et al. (2016) down to −5 MPa.

**Figure 4:**
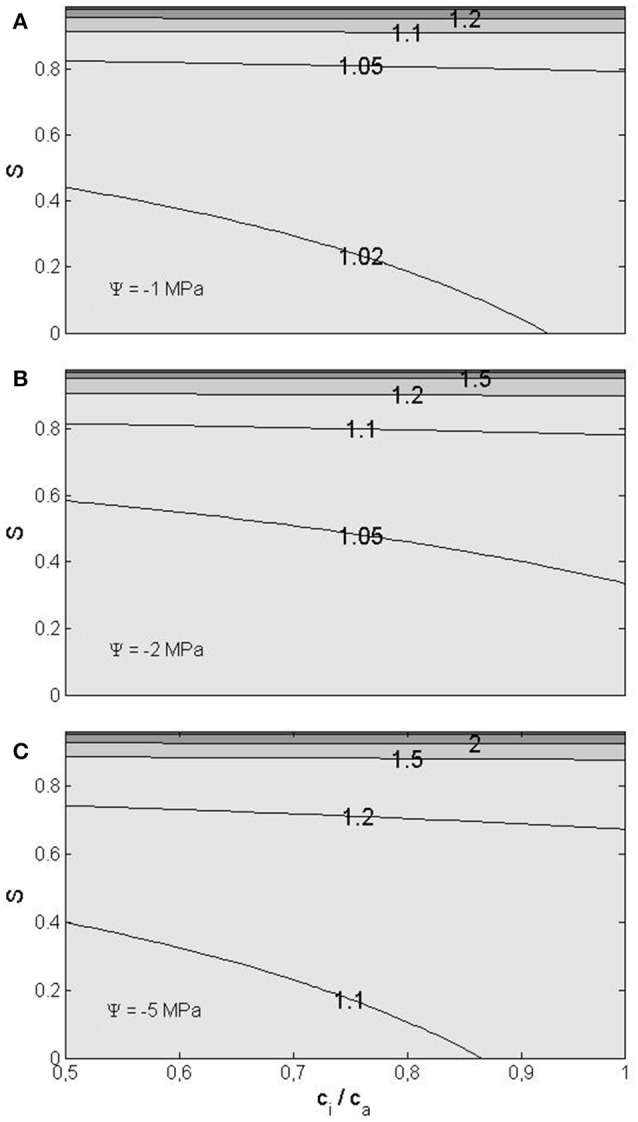
**Water use efficiency, normalized to the flat surface value, as a function of the saturation ratio ***S*** (***S*** = RH/100%) and the ratio of the internal CO_**2**_ concentration to the ambient one, for three water potential values: −1 MPa (A), −2 MPa (B), and −5 MPa (C)**.

WUE, the ratio of assimilation and transpiration rates, increases with decreasing water potential and increasing RH and internal CO_2_ concentration (Figure 4) as a result of the simultaneous decrease in transpiration rate and increase in CO_2_ assimilation rate. For example, let us consider representative conditions at temperate/boreal zone: Intermediate RH of 50%, internal CO_2_ concentration ratio of 0.7 and leaf water potential of −2 MPa. At these conditions WUE increases approximately 5% due to the decreased leaf water potential. The increase in WUE would naturally be more pronounced at lower leaf water potentials and higher RH, being 28%, if the potential was −5 MPa and RH 80% with the CO_2_ concentration ratio remaining 0.7. However, one should note that the stomatal closure ignored here would partly mask the reduced vapor pressure effect.

### Effects on the estimation of the stomatal conductance and the leaf internal CO_2_ concentration

**Figure 5:**
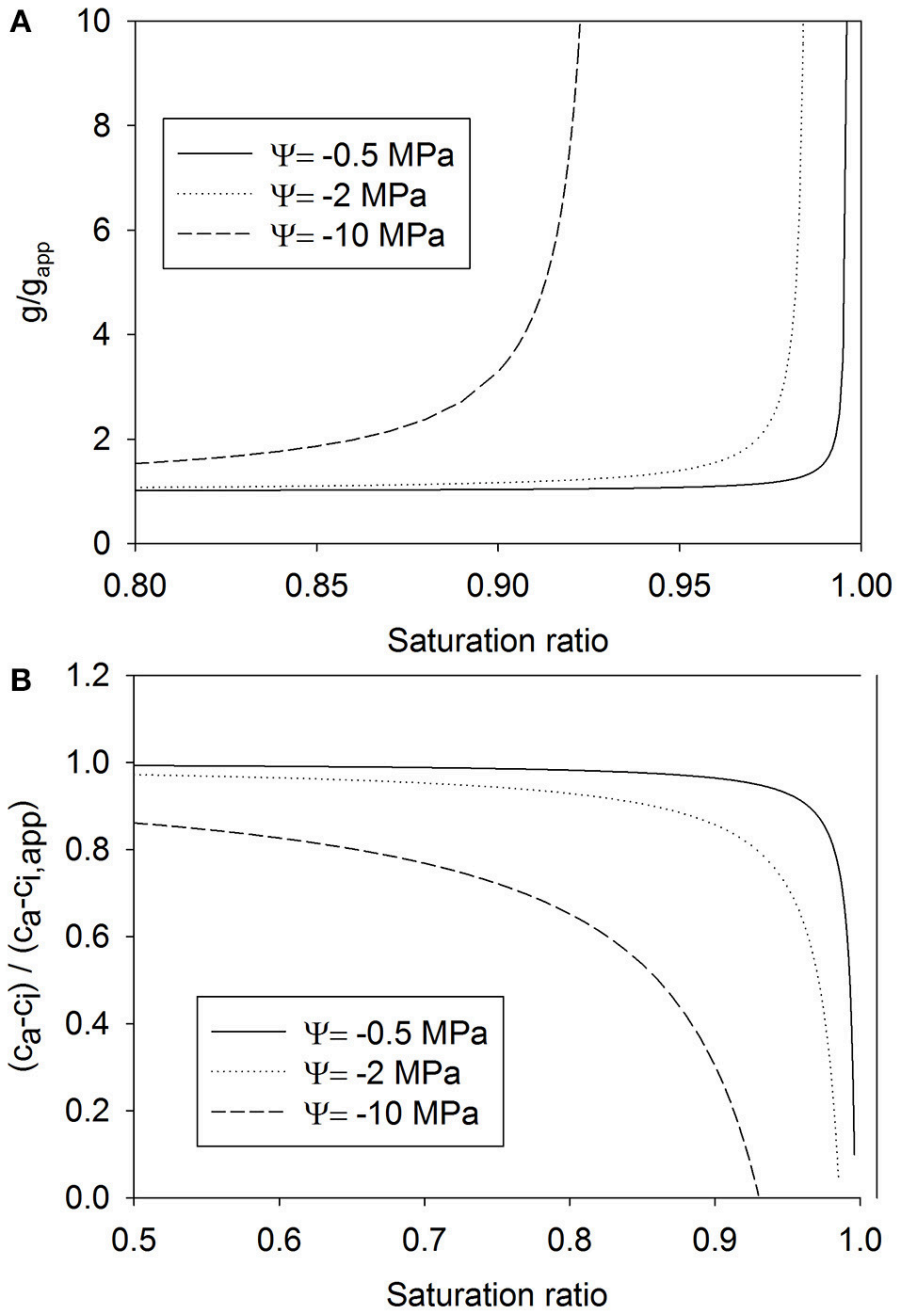
**The ratio of the actual stomatal conductance to the apparent stomatal conductance (see Equation 14) as a function of the saturation ratio ***S*** (***S*** = RH/100%) (A), and the ratio of the difference of the ambient CO_**2**_ concentration and the internal CO_**2**_ concentration to the difference against the apparent internal CO_2_ concentration as a function of ***S*** (B) (Equation 16), both for three water potential values**.

If the reduction in vapor pressure due to decreasing water potential is not taken into account, the calculation of stomatal conductance from transpiration and ambient air VPD will yield an underestimation of the actual stomatal conductance (Figure 5A). The error becomes noticeable when water potential is low and RH is high. The error in the estimation of stomatal conductance also propagates to the estimation of leaf internal CO_2_ concentration (Figure 5B). The actual value of leaf internal CO_2_ concentration is then underestimated.

### Reduced water vapor pressure and water uptake by “reverse transpiration”

**Figure 6:**
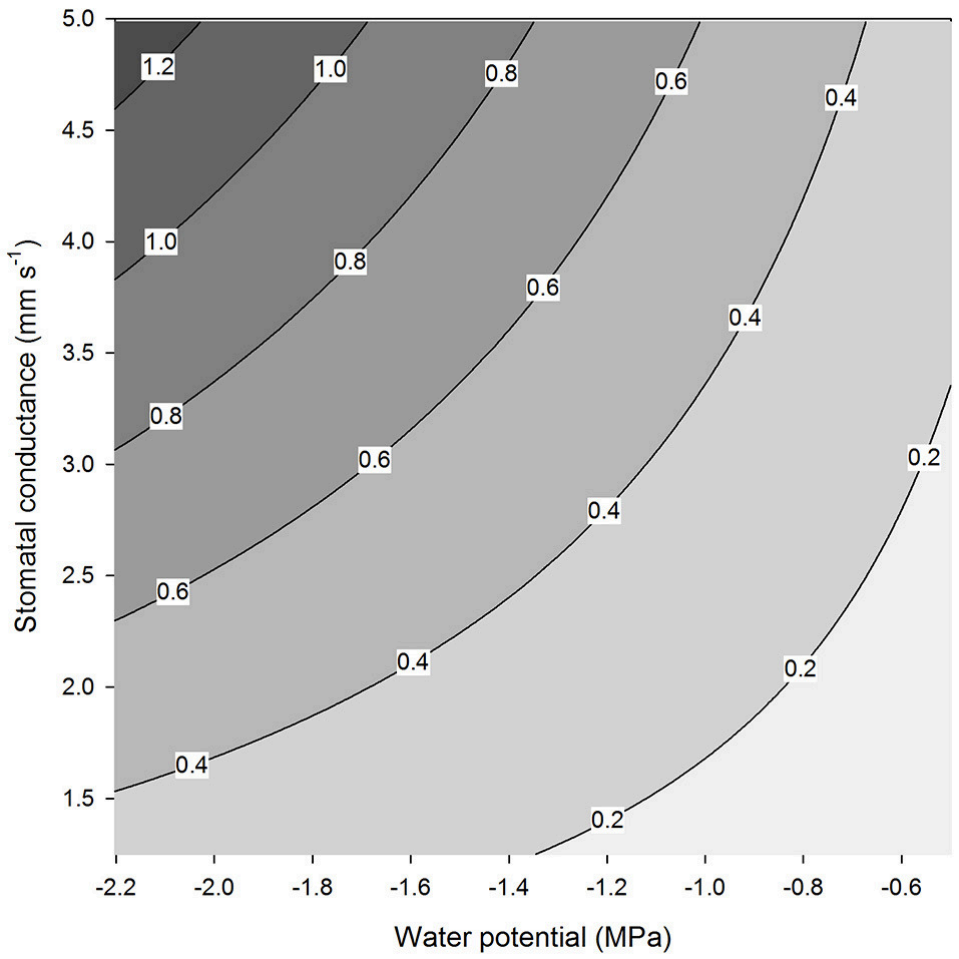
**Reverse transpiration rate (mg m^**−2**^ s^**−1**^) as a function of stomatal conductance and leaf water potential at a temperature of 20°C and a relative humidity of 100% (Equation 3)**.

We analyse the case of a coastal redwood tree (*Sequoia sempervirens*) since it grows under the conditions where water uptake by leaves is known to be significant and the relevant information is available from several references. We consider a fog event during a dry season when RH is 100% (Burgess and Dawson, 2004) and temperature is 20°C. This means that the saturation vapor concentration over a surface of water potential zero (w_i, 0_) is 0.92 mol m^−3^ (Haynes, 2010). The realistic leaf water potential values can be assumed to vary from −0.5 to −2.2 MPa (Ambrose et al., 2016). It is difficult to assess what the actual value for stomatal conductance would be during such conditions due to the difficulty in measuring the actual stomatal conductance (see Figure 5A) and also due to technical limitations of measuring small transpiration rates at high RH, when the transpiration may be also masked by condensation on the surfaces (e.g., Altimir et al., 2006). The maximum stomatal conductance for coastal redwood is likely to be around 5 mm s^−1^ (Ambrose et al., 2009). Caird et al. (2007) compiled values of nocturnal stomatal conductance measured across many different species and the largest values of c. 5 mm s^−1^ were for deciduous trees and shrubs. Increased stomatal conductance due to foggy conditions has been reported in many studies (Dawson et al., 2007; Reinhardt and Smith, 2008; Alvarado-Barrientos et al., 2015). We assume that the realistic conductance values vary from 1 to 5 mm s^−1^. By using Equation (3) with the fixed value of RH (100%) and the temperature (20°C) and the above-mentioned ranges for the water potential and stomatal conductance we obtain the range of the reverse transpiration rates shown in Figure 6. If the conductance is 2.5 mm s^−1^ and the potential −1.5 MPa, the reverse transpiration is 0.5 × 10^−3^ gm^−2^s^−1^. The reverse transpiration may be short-lived if it equilibrates the leaves. However, the obtained estimate for the reverse transpiration rate is of the same order of magnitude as the measured downwards sap flow in these trees (1.5 L h^−1^, which equals 0.6 × 10^−3^ g m^−2^ s^−1^ given a total leaf area of 660 m^2^ (Burgess and Dawson, 2004). This sap flow rate is equal to ~5–7% of maximum daytime transpiration values (Burgess and Dawson, 2004). Transpiration will remain negative as long as RH is higher than 99% given a leaf water potential of −1.5 MPa.

## Discussion

Our theoretical calculations demonstrate that the decrease in vapor pressure in the sub-stomatal cavity in the leaves due to negative water potential has a significant role in plant leaf gas exchange. It will also induce foliar water uptake through the stomata from ambient air even at RHs slightly below 100%. Although the effect of decreasing vapor pressure of water and gases with decreasing water potential is a well-recognized phenomenon in many other fields of science, its role in plant stomatal water and CO_2_ exchange has been ignored. The potential main reason for this is that its effect on stomatal gas exchange is masked by other factors and therefore, it is difficult to observe via standard gas exchange measurements. The masking factors are (1) Differences in temperature between the ambient air and the leaf will lead to a deviation of leaf internal vapor pressures from the one calculated based on the air temperature; (2) Stomata also typically close with decreasing water potential (e.g., Buckley, 2005) to prevent e.g., excess xylem embolism formation (Tyree and Sperry, 1989), and this increases WUE independently (e.g. Brodribb, 1996); (3) Low leaf water potentials can induce adverse effects on CO_2_ assimilation and WUE due to metabolic impairment of photosynthesis (Flexas and Medrano, 2002, Chaves et al., 2003) and decreased mesophyll conductance (Warren et al., 2004, Grass and Magnani, 2005) acting in the opposite direction to decrease water use efficiency during low water potentials.

Our calculations predict that the increase in WUE resulting from the decreased vapor pressures of water and CO_2_ alone is more than 5% in typical conditions of moist climates and much larger in arid regions. This result suggests that interpreting measured, drought-induced reduction in transpiration and increase in WUE directly as stomatal closure without considering the changes in vapor pressure of water and CO_2_ leads to biased conclusions about leaf gas exchange during drought. In general, the increase in the ratio of the aqueous to air phase concentration with decreasing water potential applies also to the exchange of other gases. For example, decreasing water potential would also increase the solubility of oxygen to water at the air-water interfaces, which would affect photorespiration. The solubility of volatile organic compounds to the xylem sap would also increase with decreasing water potential. The effect would be even stronger for many of the biogenic volatile organic compounds in comparison to CO_2_ since their partial molar volume is typically larger than that of CO_2_.

Water uptake via leaves can be an ecologically important water resource (Munné-Bosch et al., 1999; Caird et al., 2007; Breshears et al., 2008; Limm et al., 2009). While other mechanisms of water uptake operate simultaneously (e.g., foliar uptake through the cuticle), (e.g., Kerstiens, 1996; Eller et al., 2013), reversed transpiration by capillary condensation through stomata is based on ubiquitous connection of sub-stomatal interfaces to apoplastic water and only requires RH to be close to 100%. Therefore, it may be an efficient, naturally occurring means of water uptake for plants occupying areas of regular fogs such as the Californian coast, the Namib Desert in southern Africa, or the Chilean highlands. For example, tree structure and dynamics of stomatal control in coastal redwood in the coastal Californian forest would give good conditions for “reverse transpiration.” Coastal redwoods have one of the largest leaf area indices known and exert only weak stomatal control during nights (Burgess and Dawson, 2004), while stomatal conductance during times of CO_2_ assimilation have been found to be amongst the smallest encountered anywhere (Koch et al., 2004). Recent studies have reported foliar water uptake, and subsequent improvement of plant water status also in tropical cloud forests (Eller et al., 2013; Goldsmith et al., 2013; Gotsch et al., 2014), and in boreal forests (Berry and Smith, 2014). Water may also enter the leaves in liquid form through stomata mediated by the presence of bacteria, fungal hyphae and mucilage, or by a decrease in surface tension by aerosol deposition (Burkhardt et al., 2012). Interestingly, many species have been reported to gradually increase stomatal opening during predawn hours (Caird et al., 2007), which is exactly when RH is typically at its highest.

As we have shown by model calculations, decreasing leaf water potential lowers the vapor pressure of water, which leads to a reduction in the transpiration rate. Decreasing water potential increases the solubility of CO_2_, which leads to increasing leaf CO_2_ assimilation rate. From an ecological viewpoint, increasing WUE with decreasing water availability could benefit plants to grow in drier environmental conditions. The changes in water vapor pressure may also cause biases in the estimates of stomatal conductance and of leaf internal CO_2_ concentrations from leaf gas exchange measurements especially when RH is high and/or water potential is low. Manufactures of porometers and leaf gas exchange measurement systems should look for an option to incorporate leaf water potentials in measurement set-ups. These theoretical predictions demand further experimental study.

## Author contributions

TV designed the work together with SS, TG, EN, PH, and TH. YS participated in the interpretation of the results. TV and TH wrote the most part of the paper and all others commented it critically with important intellectual contribution. All authors approve the final version and agree for all aspects of the work.

### Conflict of interest statement

The authors declare that the research was conducted in the absence of any commercial or financial relationships that could be construed as a potential conflict of interest.
