# Supplementary material for: Effect of Leaf Water Potential on Internal Humidity and CO2 Dissolution: Reverse Transpiration and Improved Water Use Efficiency under Negative Pressure
Source: Front Plant Sci. 2017 Feb 6;8:54. doi: 10.3389/fpls.2017.00054 (PMC5292819; doi:10.3389/fpls.2017.00054)
Supplement: Supplementary file 1 [file Presentation1.PDF]

Supporting Information to

Physical mechanism of reversed transpiration and enhanced water-use efficiency under plant water stress

Algebraic manipulation: Derivation of Eqn 10

Combining Eqs. 6 and 7 gives

$$J_{CO2}^{air} = \left(c_a - \frac{c_{aq}}{H_{CO2} \exp\left(\frac{\Psi V_{CO2}}{RT}\right)} \right) g_{CO2}^{air}$$

Eqs. 8 and 9 can be combined to give

$$J_{CO2}^{aq} = (c_{aq} - \frac{A}{f}) g_{CO2}^{aq}$$

In steady state, the CO_2 fluxes in the air and aqueous phase (J_{CO2}^{air} and J_{CO2}^{aq}) and assimilation rate *A* must be equal to each other. Then, one can solve for A

$$A = \frac{c_a g_{CO2}^{air} H_{CO2} \exp\left(\frac{\Psi V_{CO2}}{RT}\right)}{H_{CO2} \exp\left(\frac{\Psi V_{CO2}}{RT}\right) + \frac{g_{CO2}^{air}}{g_{CO2}^{aq}} + \frac{g_{CO2}^{air}}{f}}$$

Under a flat air-water interphase (zero tension) *A* is

$$A_{flat} = \frac{c_a g_{CO2}^{air} H_{CO2}}{H_{CO2} + \frac{g_{CO2}^{air}}{g_{CO2}^{aq}} + \frac{g_{CO2}^{air}}{f}}$$

Further algebraic manipulation reveals that the relative increase in CO_2 assimilation rate resulting from the Kelvin effect can be expressed simply in terms of the ratio between internal (air phase) and ambient CO_2 concentration

$$\frac{A}{A_{flat}} = \frac{\dfrac{c_a \gamma_{CO2}^{air} H_{CO2} \exp\left(\frac{\Psi V_{CO2}}{RT}\right)}{H_{CO2} \exp\left(\frac{\Psi V_{CO2}}{RT}\right) + \frac{\gamma_{CO2}^{air}}{\gamma_{CO2}^{aq}} + \frac{\gamma_{CO2}^{air}}{f}}}{\dfrac{c_a \gamma_{CO2}^{air} H_{CO2}}{H_{CO2} + \frac{\gamma_{CO2}^{air}}{\gamma_{aq}} + \frac{\gamma_{CO2}^{air}}{f}}} = \frac{\exp\left(\frac{\Psi V_{CO2}}{RT}\right)\left(H_{CO2} + \frac{\gamma_{CO2}^{air}}{\gamma_{CO2}^{aq}} + \frac{\gamma_{CO2}^{air}}{f}\right)}{H_{CO2} \exp\left(\frac{\Psi V_{CO2}}{RT}\right) + \frac{\gamma_{CO2}^{air}}{\gamma_{CO2}^{aq}} + \frac{\gamma_{CO2}^{air}}{f}}$$

$$= (^{**}) \frac{\dfrac{c_{aq} \exp\left(\frac{\Psi V_{CO2}}{RT}\right)}{c_i} + \dfrac{(c_{aq} - c_c) \exp\left(\frac{\Psi V_{CO2}}{RT}\right)}{c_a - c_i} + \dfrac{c_c \exp\left(\frac{\Psi V_{CO2}}{RT}\right)}{c_a - c_i}}{\dfrac{c_{aq} \exp\left(\frac{\Psi V_{CO2}}{RT}\right)}{c_i} + \dfrac{(c_{aq} - c_c)}{c_a - c_i} + \dfrac{c_c}{c_a - c_i}}$$

$$= \frac{\dfrac{c_{aq} \exp\left(\frac{\Psi V_{CO2}}{RT}\right)}{c_i} + \dfrac{c_{aq} \exp\left(\frac{\Psi V_{CO2}}{RT}\right)}{c_a - c_i}}{\dfrac{c_{aq} \exp\left(\frac{\Psi V_{CO2}}{RT}\right)}{c_i} + \dfrac{c_{aq}}{c_a - c_i}}$$

$$= \frac{c_a c_i (c_a - c_i) \exp\left(\frac{\Psi V_{CO2}}{RT}\right)}{c_i (c_a - c_i)\left((c_a - c_i) \exp\left(\frac{\Psi V_{CO2}}{RT}\right) + c_i\right)} = \frac{c_i \exp\left(\frac{\Psi V_{CO2}}{RT}\right)}{(c_a - c_i) \exp\left(\frac{\Psi V_{CO2}}{RT}\right) + c_i}$$

$$= \frac{1}{1 + \dfrac{c_i}{c_a \exp\left(\frac{\Psi V_{CO2}}{RT}\right)}\left(1 - \exp\left(\frac{\Psi V_{CO2}}{RT}\right)\right)} = \frac{1}{1 + \dfrac{c_i}{c_a}\left(\dfrac{1}{\exp\left(\frac{\Psi V_{CO2}}{RT}\right)} - 1\right)}$$

$$= \left[\frac{c_i}{c_a}\left(\frac{1}{\exp\left(\frac{\Psi V_{CO2}}{RT}\right)} - 1\right) + 1\right]^{-1}$$

(**) $H_{CO2} = \frac{c_{aq}}{c_i}$

$\frac{g_{CO2}^{air}}{g_{CO2}^{aq}} = \frac{c_{aq} - c_c}{c_a - c_i}$ from Eqs. 7 and 8

$\frac{g_{CO2}^{air}}{f} = \frac{c_c}{c_a - c_i}$ from Eqs. 7 and 9

Calculation of the transient time for CO_2 equilibrium to be reached between the air and water phases in the leaf.

The amount of CO_2 (n_{CO2}) dissolved in a unit surface area of a leaf of 1 mm in thickness (assuming its 100% water) is typically of the order of

$$n_{co2} = c_{CO2}^{sat} V_{water} = 0.015 \frac{mol}{m^3} \times 1^{-3} m = 15 \times 10^{-6} mol\ m^{-2}$$

where V_{water} is the amount of water per unit leaf area. A decrease in leaf water tension from 2 MPa to 0 would lead to a 2.5 % increase in the air phase CO_2 concentration (see Eq 6), which would release 2.5 % of the CO_2 from the water phase to the air phase in the leaf. Given a typical CO_2 assimilation rate of $10 \cdot 10^{-6}$ mol m^{-2} s^{-1}, the sudden release in CO_2 from the water phase due to the tension release would equal to the amount CO_2 assimilated in

$$t = \frac{0.025 \times 15 \times 10^{-6} mol m^2}{10 \times 10^{-6} mol m^2 s^1} = 0.04 s$$

In other words, a new steady state in leaf water CO_2 concentration after an instantaneous change in water tension would be reached in approximately 0.04 s. Since we are dealing time scales much longer than this in CO_2 assimilation rate, the steady state approach used in our calculations is well justified.
