# Supplementary material for: Effect of Leaf Water Potential on Internal Humidity and CO2 Dissolution: Reverse Transpiration and Improved Water Use Efficiency under Negative Pressure
Source: Front Plant Sci. 2017 Feb 6;8:54. doi: 10.3389/fpls.2017.00054 (PMC5292819; doi:10.3389/fpls.2017.00054)
Supplement: Supplementary file 2 [file Presentation2.pdf]

Appendix A. The reduction of water vapour pressure; the Kelvin equation and Raoult's law

The water vapour concentration (w_i) over an air-aqueous solution interface with a curvature (r) in relation to the water vapour concentration over a flat surface of pure water ($w_{i,0}$) can be expressed as (Seinfeld and Pandis 1998)

$$w_i = w_{i,0} x_{H2O} \exp\left(-\frac{2\gamma V_{H2O}}{rRT}\right) \qquad \text{(A1)}$$

where x_{H2O} is the mole fraction of water in the aqueous solution, γ is surface tension of water, V_{H20} is the molar volume of water, r is the radius of curvature (defined positive for a concave surface), R is the universal gas constant and T is the interfacial temperature. The first term (x_{H2O}) is from Raoult's law and it represents the decrease in water vapour concentration due to dilution of the aqueous solution, i.e. it is the osmotic term of the water potential. The second (exponential) term is the Kelvin equation and it represents the decrease in water vapour concentration due to the curvature of the meniscus.

The decrease in vapour pressure above the concave surfaces in comparison to flat surfaces, known also as the Kelvin effect, was formulated already in 1871 (see e.g. Seinfeld and Pandis (1998) for a detailed description of the phenomenon). The Kelvin effect results from molecules on a flat surface experiencing a different net attraction to their neighboring molecules compared to the molecules on a curved surface (Fig. A1). Because vapour pressure is inversely related to the energy required for removing a molecule from the surface (Clausius-Clapeyron equation see e.g. Seinfeld and Pandis (1998)), the vapour pressure of the liquid and dissolved gases is reduced over a concave liquid-gas surface, and elevated over a convex surface. In many other fields the Kelvin effect is well documented, experimentally confirmed (Reiss and Koper 1995, Pera-Titus *et al.* 2009, Nanda *et al.* 2002) and widely accounted for. For example, it causes condensation of water in small capillaries and porous media (Fisher and Israelachvili 1979) and in atmospheric sciences it has important consequences for the dynamics of small water droplets (Vesala *et al.* 1997).

If we further apply Young-Laplace's formula (Nobel 2005)

$$T_N = \frac{2\gamma}{r} \qquad \text{(A2)}$$

where T_N is the water tension, the equation (A1) can be written as

$$w_i = w_{i,0} x_{H2O} \exp\left(\frac{-T_N V_{H2O}}{RT}\right) \qquad \text{(A3)}$$

Note that the wettability of the capillaries containing the water-air interface, i.e. the contact angle between the capillary and water, will not influence the relation between water tension and radius of curvature. However, the wettability of the capillaries (defined by the contact angle θ) will affect the maximum pore size (r_{mp}) which is able to support the capillary column of water under tension

so that $r_{mp} = r \cos\theta$ (Nobel 2005). As tension increases, water will withdraw totally from those pores that are too large and/or non-wettable to support the water column.

This relation can be also written as a function of water potential only (as used in the calculations in the main text). The water potential is the same in the liquid and gaseous phase, and is expressed as (Nobel 2005)

$$\Psi = \frac{\mu - \mu_0}{V_{H2O}} = \frac{RT}{V_{H2O}} \ln \frac{w_i}{w_{i,0}} \qquad \text{(A4)}$$

where μ is the chemical potential of water and μ_0 is the chemical potential of water at reference state of atmospheric pressure. Solving for w_i leads to

$$w_i = w_{i,0} \exp\left(\frac{\Psi V_{H2O}}{RT}\right) \qquad \text{(A5)}$$
